# Association of Endogenous Retroviruses and Long Terminal Repeats with Human Disorders

**DOI:** 10.3389/fonc.2013.00234

**Published:** 2013-09-11

**Authors:** Iyoko Katoh, Shun-ichi Kurata

**Affiliations:** ^1^Department of Microbiology, Interdisciplinary Graduate School of Medicine and Engineering, University of Yamanashi, Chuo, Yamanashi, Japan; ^2^Redox Response Cell Biology, Medical Research Institute, Tokyo Medical and Dental University, Tokyo, Japan

**Keywords:** human endogenous retrovirus, HERV, long terminal repeat, LTR, cancer, retrotransposon, autoimmune

## Abstract

Since the human genome sequences became available in 2001, our knowledge about the human transposable elements which comprise ∼40% of the total nucleotides has been expanding. Non-long terminal repeat (non-LTR) retrotransposons are actively transposing in the present-day human genome, and have been found to cause ∼100 identified clinical cases of varied disorders. In contrast, almost all of the human endogenous retroviruses (HERVs) originating from ancient infectious retroviruses lost their infectivity and transposing activity at various times before the human-chimpanzee speciation (∼6 million years ago), and no known HERV is presently infectious. Insertion of HERVs and mammalian apparent LTR retrotransposons (MaLRs) into the chromosomal DNA influenced a number of host genes in various modes during human evolution. Apart from the aspect of genome evolution, HERVs and solitary LTRs being suppressed in normal biological processes can potentially act as extra transcriptional apparatuses of cellular genes by re-activation in individuals. There has been a reasonable prediction that aberrant LTR activation could trigger malignant disorders and autoimmune responses if epigenetic changes including DNA hypomethylation occur in somatic cells. Evidence supporting this hypothesis has begun to emerge only recently: a MaLR family LTR activation in the pathogenesis of Hodgkin’s lymphoma and a HERV-E antigen expression in an anti-renal cell carcinoma immune response. This mini review addresses the impacts of the remnant-form LTR retrotransposons on human pathogenesis.

## Human Endogenous Retroviruses and Mammalian Apparent LTR Retrotransposons are Long Terminal Repeat-Retrotransposons in the Human Genome

Endogenous retroviruses (ERVs) stem from ancient infectious retroviruses that were reverse transcribed and integrated into the germ cell chromosomal DNA of ancestral host animals. The integrated viral genomes, termed proviruses, were subsequently fixed, and have been inherited for tens of millions of years (Myr). “Human endogenous retroviruses” (HERVs) refer to human ERVs, most of which appear to have entered the genome after the separation of Old and New World monkeys (∼35 Myr ago), and have increased in copy numbers through the human/primate evolution (1–4). The present-day human genome contains ∼400 thousand copies of HERVs and related sequences, accounting for ∼4.6% of the ∼3 billion nucleotide sequences (5, 6). In the eras of their copy number expansion, HERVs in the original form of 5′LTR-*gag*-*pro*-*pol*-*env*-3′LTR amplified themselves autonomously. However, all HERVs in the present-day humans are replication-incompetent, having varying degrees of mutations and deletions. Despite the absence of an obvious transposing activity, HERVs are classified as transposons based on the evidence of transposition during the human evolution and the existence of numerous animal ERVs presently mobilizing in their host genomes as represented by mouse intracisternal A particles (7, 8).

Human endogenous retroviruses are divided into 50–200 families primarily depending on the primer binding site (PBS) sequences complementary to the 3′ end of a cellular tRNA (9–11). For example, HERV-K and -W indicate utilization of tRNA^Lys^ and tRNA^Try^ as the primers for reverse transcription, respectively. This terminology has nothing to do with their histories or functions. Nevertheless, the HERV-F/H family represents the oldest HERVs which entered into our genome before the split of simians and prosimians ∼60–70 Myr ago (12), while the HERV-K (HML-2) family contains the youngest HERVs having the best-preserved retroviral open reading frames (ORFs) (13, 14).

In addition to HERVs, the long terminal repeat (LTR) retrotransposon class in humans have another big subclass of “mammalian apparent LTR retrotransposons” (MaLRs), which consists of ∼240,000 copies accounting for 3.65% of the genomic sequences (5) (Table 1). Despite the classification, MaLRs lack a PBS and *gag*-*pro*-*pol* genes shared by animal retroelements (*Copia*/*Ty1*, *Gypsy*/*Ty3*, etc.) and retroviruses, and are thought to have originated before the radiation of eutherian mammals, 80–100 Myr ago (15, 16). The simple 5′LTR-ORF-3′LTR structure without a *pol* gene encoding reverse transcriptase (RT) and integrase suggests non-autonomous transposition. The transposon-like human element-1 (THE1) is known as a most widespread MaLR family in primates.

**Table 1:** **Transposons in the human genome and their influences on health and diseases**.

Classification	Class II	Class I			
	DNA transposons	Retrotransposons			
		LTR retrotransposons		Non-LTR retrotransposons	
Members (28, 83)	Super families of MITE piggyBac, Merlin, mutator, etc.	Human endogenous retroviruses (HERVs/ERVs)	Solitary LTRs	LINEs (L1, L2, L3)	SINEs (Alu), SVAs, B1s pseudogenes
		Mammalian apparent LTR retrotransposons (MaLRs)	
Mechanisms of transposition or replication (28, 83)	Autonomous, “cut and paste”	HERV, autonomous, “copy and paste” (13): (1) retrotransposition, (2) reinfection (complementation *in trans*)		Autonomous, “copy and paste” (17)	Non-autonomous, Transposition in *trans* by L1 (18, 19)
		MaRL, non-autonomous (15)	
Reverse transcriptase (83)	(−)	HERV(+)	(−)	(+)	(−)
		MaLR(−)	
Number of copies in the genome (×1,000) (5)	294	443 (ERV-class I, 112; class II, 8; class III, 83; Ma LR, 240)		868 (L1, 516)	1558 (SVA, 2.76; Alu, 1,090)
Percentage (%) of the genome (5)	2.84	8.29 (HERV, 4.64; MaLR, 3.65)		20.42	13.14
Structural integrity	(−)	(−)	(+)/(−)	L1 (+)	(+)/(−)
Transposing activity in present-day humans (28)	Inactive	Very limited or none (13, 35)		Active (L1 only)	Active with L1
Time of transposition in human development		None reported		Germline, embryo, and somatic cells (28)	
Recent transpositions/insertions in evolution	∼37 Myr ago	8–17 HERV-K insertion the past ∼6 Myr (31)	Not specified	>7500 Transpositions in the past 6 Myr (23)	
Impacts of the identified elements on human health and homeostasis		Placenta development	Alteration of cellular gene expression by LTR insertion (∼2 5 loci experimentally characterized) (45, 53)	Gene family generated by SVA through transduction (*AMAC*, ∼7–14 Myr ago)	
		(*Syncytin-1*/*ERVW-1*) (55, 56)	
		(*Syncytin-2*/*ERVFRD-1*) (57)		Modulation of gene expression by Alu through RNA editing (*APOBEC3G*, *F11R*, etc.) (19)	
Transcriptional activation		Hypomethylation (81)		Hypomethylation (27)	
		*Trans*-activation (37)		*Trans*-activation (SRY, RUNX3, YY1)	
Disease-related mechanisms (28, 83)		Transcriptional activation of neighboring gene		Insertional mutagenesis by transposition	
		Viral gene expression		Transduction by transposition	
		Recombination (hypothesized)	
Related disorders	None reported	Hodgkin’s lymphoma (91) (activation of *CSF1R* by a THE1B LTR)		∼100 Reported cases by transposition (columns below) (27, 28, 83)	
				Hypomethylation detected in malignancy	
		Renal cell carcinoma (89, 90) (HERV-E CT-RCC-8/9, target antigen of CD8+ cells)		
		Other tumors (6, 68) (expression of HERVs)		
		Autoimmune disorders including rheumatoid arthritis, multiple sclerosis, Sjögren’s, and psoriasis (92, 100, 101) (expression of HERVs)	
		Facioscapulohumeral dystrophy (108) (THE1D LTR activation by DUX4)	
				Hemophilia (*FVIII*, *FIX*), duchenne muscular dystrophy (*DMD*), neurofibromatosis type I (*NF1*), tumors, etc.	Hemophilia (*FVIII*, *FIX*), breast cancer (*BRCA2*), neurofibromatosis type I (*NF1*), etc.

## Mobilization of Non-LTR Retrotransposons

Transposons currently mobilizing on the human chromosomes include the long interspersed elements (LINEs), short interspersed elements (SINEs), and “SINE-R, variable of number of tandem repeats (VNTRs) and Alu” (SVA) elements, all of which are in the “non-LTR retrotransposons” family. L1 elements in the LINE family, are the only active autonomous retrotransposons, and replicate by a simple “copy and paste” mechanism involving target-site primed reverse transcription (17). *Alu* elements, the youngest subtypes of SINEs, and SVAs are apparently mobilized *in trans* by L1s (18, 19). Retrotransposition of these elements occurs in germline, early embryonic development, and in somatic cells (20–22).

In the past 6 Myr, >7,500 transpositions occurred in the primate genome (23). One retrotransposition per 10–100 births is estimated (19, 24). Thus, L1, Alu, and SVA elements have substantially impacted the human genome organization in evolution (25). Furthermore, the non-LTR retrotransposons are causative agents of human genetic disorders (26, 27); ∼100 cases of disorders caused by non-LTR retrotransposon insertions have been reported (27, 28) (Table 1).

## HERVs and MaLRs Exist as Non-Transposable Elements

In comparison with the intra-nuclear transposition mechanisms of non-LTR retrotransposons, both of the ERV amplification pathways, “retrotransposition,” and “reinfection,” are tricky. The former comprises multiple steps including particle-like assembly in the cytosol, reverse transcription, nuclear translocation, and chromosomal insertion. The latter requires infectious virus particle production followed by germ-line infection ultimately leading to chromosomal integration and fixation (13, 29, 30). Defective ERVs are also able to increase their copy numbers by “complementation in *trans*,” where a matching element at a different locus serves as the helper (13, 29). These mechanisms underlay the HERV amplification in the past.

However, no evidence of HERV transposition has been detected in present-day humans, either in a scientific or clinical background. Studies suggested 8–17 full-length HERV-K insertions after the divergence of humans from chimpanzees ∼6 Myr ago (31, 32). The insertion of HERV-K113 and -K115 were estimated to be a minimum 0.8–1 Myr ago, and that of HERV-K106 to be ∼150,000 years ago (33, 34). Even though the possibility of the youngest HERV-K mobilization still remains (35), HERVs as transposable elements do not impose a “clear and present danger” to humans. In addition, transposition of the older resident MaLRs is the most improbable.

## Hundreds of Thousands of LTRs in the Genome Can Provide Binding Sites for Transcription Factors

Solitary LTRs, or solo LTRs, were generated from existing HERVs and MaLRs by the loss of internal sequences upon recombination between the 5′ and 3′ LTRs within the same copies or between separate LTRs. The human genome contains ∼50 copies of ERV-9 provirus forms and 3,000–4,000 copies of ERV-9 solitary LTRs (36). A recent study identified 944 HERV-K (HML-2) solitary LTRs versus 91 copies of full-length and nearly full-length HERV-K (HML-2) (14). In general, 85% of the HERV copies consist of solitary LTRs (5). The majority of MaLRs also exist as solitary LTRs (15).

Retrovirus LTRs (300–1300 bp) in the U3–R–U5 architecture are essential for high-level expression of the viral sequences (37). The U3 region contains a TATA box and transcription regulatory sequences which govern ubiquitous or tissue-specific gene expression. For instance, the U3 regions of human immunodeficiency viruses (HIVs) and the group of human T-cell leukemia viruses (HTLVs) have NF-κB and CREB/ATF binding sequences, respectively (38–41). Regulatory factors bind to the cognate *cis*-acting sequences to induce transcription from the initiation site corresponding to the 5′ terminus of the R region. In fact, the HERV-K (HML-2) LTR has Sp1/Sp3 and microphthalmia-associated transcription factor-M (MITF-M) binding sites in U3 (42, 43).

Therefore, the ∼400,000 copies of the LTR-retroelements scattered throughout the human chromosomes potentially provide extra enhancer-promoter sequences and initiation sites for neighboring cellular genes. By utilization of a nearby LTR as its alternative transcription machinery, one gene can give rise to new isoforms, enhance its transcription, and alter the tissue-specificity. In this context, HERVs and MaLRs could have significantly influenced the human genome evolution (44).

## Influence of LTR/HERV Insertions on Cellular Gene Expression in Human Evolution

More than 25 experimentally characterized cellular genes show LTR-mediated evolutionary changes, in which inserted LTRs act as alternative promoters to provide new tissue-specificity, play as the major promoters, or exert only minor effects (45). For example, an ERV-L LTR plays as an alternative promoter of β1,3-galactosyltransferase 5 gene (*B3GALT5*) to induce colon-specific transcription (46). A HERV-K(HML-5) LTR serves as the major promoter of *INSL4*, a primate-specific insulin-like growth factor gene expressed in placenta (47). A HERV-E family LTR plays as an alternative tissue-specific promoter of the endothelin B receptor (*EDNRB*) gene, by which the gene expression increased ∼15% in placenta (48). LTR-derived promoters often increase placenta-specific gene expression, although the overall effect of the LTR insertion appears moderate in many cases (45). Notably, the 5′ terminus of a HERV-E insert in reversed orientation provides a salivary-specific enhancer for the human-only amylase gene *AMY1C* (49, 50).

The LTR of ERV-9 located in the β-globin locus control region (β-LCR) serves as an additional enhancer region for the downstream globin genes (36, 51). The U3 region has 5–17 tandem repeats of 37–41 bases with GAGA, CCAAT, and CCACC motifs, in which the strong affinity of NF-Y, a ubiquitous transcriptional regulatory factor, to the CCAAT motif allows a robust enhancer complex formation with other proteins (52). An ERV-9 LTR upstream of *TP63* (p63), a member of the tumor suppressor *TP53* (p53) gene family, acts as a strong promoter to express novel p63 isoforms in the testis of *hominidae* (53). Thus, some of the 5,000 copies of ERV-9 LTRs, 15–38 Myr after their integration (54), are involved in adjacent host gene control.

A HERV-W-derived Env glycoprotein encoded by *ERVW-1* (7q21.2) (also termed *syncytin-1* or *ERVWE1*) causes cytotrophoblast fusion, suggesting an essential function in syncytiotrophoblast formation in placenta tissue development (55). In addition, a HERV-FRD Env protein encoded by *ERVFRD-1*, or *syncytin-2* (6p24), was found to have an immunosuppressive activity (56), while a role in placenta morphogenesis was also suggested (57). In conjunction with the upstream regulatory sequences, the HERV-W LTR drives the strong *ERVW-1* expression in placenta (58).

## The Hypothesis of HERV-Mediated Oncogenesis

Animal exogenous retroviruses carrying oncogenes can trigger transformation of the host cells by expression of the viral oncogenes (59). The virus *env* gene can act like an oncogene (60, 61). Retroviruses without an oncogene are also oncogenic. If retroviral integration occurs in the vicinity of a proto-oncogene which typically encodes a growth factor receptor, signaling molecule, or transcriptional activator, the LTR can activate it. “Transduction” of oncogenes and “insertional mutagenesis” by the LTRs represent major retroviral tumorigenesis mechanisms (59, 62). Based on the knowledge of retroviral functions, contribution of HERVs and solitary LTRs to human tumorigenesis has long been hypothesized.

The *Np9* and *Rec* genes overlapping the *env* gene of HERV-K are particularly intriguing as candidate oncogenes. HERV-K was found as an endogenous retrovirus expressed in teratocarcinoma cell lines in an earlier study (63). Nuclear protein Np9 interacts with E3 ubiquitin ligase Ligand of Numb Protein-X (LNX), and thereby possibly influences the Notch pathway (64). *Rec* transgenic mice manifest neoplastic changes in the testes which correspond to the precursor phenotype of seminoma in humans (65). The Rec protein enhances androgen receptor-mediated transcription (66). High-level expression of Rec and Np9 also occurs in melanoma (67).

Activation of specific or selective HERVs occurs in various tumors and cell lines (68). HERV-K has been most extensively analyzed in cancers from different tissue origins. For example, HERV-K mRNA and proteins were detected in melanoma tissues and cell lines (69, 70). A recent study identified 24 HERV-K (HML-2) loci being transcribed in melanoma tissues (67). The RT and its enzyme activity increase in breast cancer tissues and hormone-treated cell lines (71). A large population (>85%) of breast cancer patients express HERV-K Env proteins which elicit both serologic and cell-mediated immune responses (72). HERV-K virus-like particle production was observed in teratocarcinoma cell lines (73, 74). HERV-K Gag and Env proteins are expressed in germ cell tumors, and the patients produce antibodies against these viral proteins (75, 76). Forward and reverse transcripts of several HERV-K loci are detected in prostate cancer cell lines (77). Furthermore, the *gag* gene of a HERV-H locus on chromosome Xp22 is frequently expressed in colon cancer tissues (78). Ovarian cancer tissues produce the Env proteins of HERV-K, ERV3, and HERV-E (79).

## Transcriptional Activation of HERVs by Epigenetic Changes in Cancers

Transposons undergo long-term epigenetic silencing by DNA methylation, which is stably maintained from gametes to early embryos and the adult tissues (80). Cancer-associated hypomethylation, or derepression, frequently occurs in repeated DNA sequences including heterochromatic DNA repeats, dispersed retrotransposons, and endogenous retroviral elements (81). Although genome wide hypomethylation in cancers seems to be a side effect of carcinogenesis rather than the cause (82), site-specific hypomethylation may possibly cause re-activation of tumor-related genes leading to initiation and progression of malignancies and other diseases (83).

Recent results show that increased HERV-K (22q11.23) expression in prostate and ovarian cancers is associated with hypomethylation of the HERV-K locus (84, 85). Reduced methylation of CpG dinucleotides within the promoter region corresponds to HERV-W expression in ovarian cancer (86) and HERV-H (Xp22.3) expression in colon, gastric, and pancreatic cancers (87). Hypomethylation of the U3 region of several HERV-W loci seems to be critical for the HERV-W activation in testicular cancer (88).

## Identification of LTRs that Contribute to Tumor Immunity and Oncogenesis

A pioneering study identified a HERV-encoded peptide as a tumor-specific antigen that works in the successful hematopoietic stem cell transplantation for the therapy of metastatic renal cell carcinoma (RCC) (89). The tumor antigen, CT-RCC-1, recognized by RCC-specific CD8+ T cells is encoded by novel spliced variants of the HERV-E transcript from chromosome 6q. Furthermore, this antigen expression results from hypomethylation of the LTR sequences, as well as hypoxia-inducible transcription factor (HIF-2α) binding to the HIF responsive element (HRE) in the LTR (90). HIF-2α is stabilized in many cases of RCC due to the von Hippel–Lindau (*VHL*) tumor suppressor gene inactivation. This whole scenario enlightens the relevance of HERVs in cancer biology and treatment.

A study on tumorigenesis of Hodgkin’s lymphoma provided the first and so far only evidence that endogenous LTR activation can be oncogenic (91). These malignant cells strongly express *CSF1R*, the colony stimulating factor 1 receptor gene, to allow CSF1/CSF1R-dependent cell growth. Intriguingly, a LTR of THE1B (a MaLR family LTR retrotransposon) is located 6.2 kb upstream of the CSF1R coding sequences, and is activated by the loss of CpG methylation to drive the aberrant *CSF1*R gene expression from the initiation site within the LTR. The regulatory region contains Sp1, GATA, AP-1, and NF-κB binding motifs. Anaplastic large cell lymphomas also have the same *CSF1R* transcript. Thus, hypomethylation of the THE1B LTR causes the *CSF1R* oncogene activation, reminiscent of “insertional mutagenesis” by exogenous retroviruses.

## Possible Contribution of HERVs to Autoimmune Diseases

Human endogenous retroviruses have also been implicated in autoimmune diseases including rheumatoid arthritis (RA), multiple sclerosis (MS), and systemic lupus erythematosus (SLE). Expression of multiple ERVs was detected in RA patients (92), and that of HERV-K10 in juvenile RA (93). A recent report indicated a strong linkage between the MS-associated retroviral element (MRSV)-type HERV-W and MS: the env RNA and protein expression significantly increases in the blood and brain cells of MS patients (94). HERV-H/F family HERV-Fc1 expression in T lymphocytes and the plasma HERV-Fc1 RNA level also increase in patients with MS (95). HERV-K18 (chromosome 1) was found to be a risk factor of MS (96) and other autoimmune disorders (97, 98). Expression of HERV-W, K, E, and a newly identified ERV-9/HERV-W variant in psoriasis was also observed (99).

Three mechanisms have been proposed for HERV-related pathology of autoimmune diseases: molecular mimicry, superantigen production, and LTR-mediated alterations of gene expression (100). The Gag and Env proteins of HERV-K10 share the peptide sequences with the rheumatoid factor epitopes on IgG1Fc, implying molecular mimicry (101). The *env* genes of defective HERV-K alleles at 1q21.2–q22 encode superantigens, and are transcriptionally activated by the interferon α signaling to cause polyclonal T-cell activation (102, 103). There was an interesting hypothesis that solitary LTRs in the 5′-flanking region of the *HLA-DQB1* gene (encoding major histocompatibility complex, class II, DQ beta 1) influence the susceptibility to autoimmune diseases (104–106). However, these results remain ambiguous, and need to be more critically examined to determine whether or not the LTR/HERV activation is the true cause of the clinical consequences (101, 107).

Furthermore, the LTR of MaLR family THE1D is activated by transcription factor DUX4 which is specifically expressed in facioscapulohumeral dystrophy, implying involvement of DUX4 in the pathophysiology (108). Hypomethylation of HERV-E and HERV-K was also observed in SLE patients (109).

## Concluding Remarks

Human endogenous retroviruses are remnant forms of infectious retroviruses that integrated into the chromosomal DNA of germ-line cells of human ancestors, increased their copy numbers and have been inherited by present-day humans. None of the HERVs poses an immediate risk as a transposable element. However, solitary LTRs derived from HERVs and MaLRs dominate the provirus forms in the copy numbers, and can serve as redundant enhancer-promoter sequences for nearby cellular genes. When the DNA methylation-mediated suppression system becomes compromised, HERVs and LTRs can cause detrimental and/or self-protecting effects. Two prominent examples of the clinically significant HERV/LTR activation have been reported: CSF1R oncogene activation by a MaLR LTR in Hodgkin’s lymphoma and RCC-specific novel HERV-E antigen expression facilitating the immunotherapy. Future researches in oncology and immunogenetics will unveil more details about the endogenous LTR functions in human pathogenesis.

## Conflict of Interest Statement

The authors declare that the research was conducted in the absence of any commercial or financial relationships that could be construed as a potential conflict of interest.
